# The Prenatal Hormone Milieu in Autism Spectrum Disorder

**DOI:** 10.3389/fpsyt.2021.655438

**Published:** 2021-07-01

**Authors:** Whitney Worsham, Susan Dalton, Deborah A. Bilder

**Affiliations:** ^1^University of Utah School of Medicine, Salt Lake City, UT, United States; ^2^Department of Obstetrics and Gynecology, University of Utah, Salt Lake City, UT, United States; ^3^Division of Child & Adolescent Psychiatry, Department of Psychiatry, University of Utah, Salt Lake City, UT, United States

**Keywords:** autism, autism spectrum disorders, steroid hormone (progesterone, testosterone, estradiol), perinatal, prenatal, risk factors

## Abstract

Though the etiology of autism spectrum disorder (ASD) remains largely unknown, recent findings suggest that hormone dysregulation within the prenatal environment, in conjunction with genetic factors, may alter fetal neurodevelopment. Early emphasis has been placed on the potential role of *in utero* exposure to androgens, particularly testosterone, to theorize ASD as the manifestation of an “extreme male brain.” The relationship between autism risk and obstetric conditions associated with inflammation and steroid dysregulation merits a much broader understanding of the *in utero* steroid environment and its potential influence on fetal neuroendocrine development. The exploration of hormone dysregulation in the prenatal environment and ASD development builds upon prior research publishing associations with obstetric conditions and ASD risk. The insight gained may be applied to the development of chronic adult metabolic diseases that share prenatal risk factors with ASD. Future research directions will also be discussed.

## Introduction

Autism spectrum disorder (ASD) is a neurodevelopmental disorder characterized by persistent deficits in social interaction and communication in addition to stereotyped, repetitive behaviors (1). While the etiology of ASD remains largely unknown, multifactorial contributors such as genetics, neuroanatomical abnormalities, and the environment likely play a role (2). Prenatal factors associated with elevated ASD risk include maternal conditions that contribute to a suboptimal prenatal environment, particularly as it relates to inflammation, metabolism, and steroid hormone regulation (3, 4).

Prenatal stress (5, 6), maternal immune dysfunction (5, 7–9), pre-existing/gestational diabetes (3, 10), pre-pregnancy obesity (11), weight gain during pregnancy (12), pre-existing/gestational hypertension (13, 14), polycystic ovarian syndrome (PCOS) (4, 15) and prenatal complications such as low birth weight (3) and pre-term birth (16) have all been associated with ASD in offspring. How these conditions may alter prenatal mechanisms promoting ASD pathogenesis has yet to be clearly established, though several physiological processes that link these conditions have been implicated including direct insults (e.g., oxidative stress, hypoxia, inflammation) and adaptive responses (e.g., epigenetic changes, fetal programming) (17–20).

Studying the relevance of prenatal risk factors in ASD pathogenesis inherently involves understanding the remarkable, well-coordinated interaction among the mother, fetus, and placenta—referred to as the maternofetoplacental unit (21, 22). The interdependence across the maternofetoplacental unit for steroid hormone production, immune response mediation, and nutrient transfer is particularly relevant to sustain pregnancy and ensure newborn viability. The placenta, together with the amnion and chorion, create a unique immune environment that permits co-existence of the fetal allograft within the mother while accessing her rich nutrient and oxygen supply for development, growth, and survival.

Immune response and steroid production are intrinsically linked during pregnancy with the placenta acting as the key mediator, particularly regarding estradiol and progesterone synthesis. Estradiol augments both cell- and antibody-mediated immune responses (23). Rising levels of estradiol during pregnancy promote an immunologic shift from an inflammatory state to a regulatory response (23–26). The dramatic increases in progesterone enhance maternal-fetal tolerance (27) and promote anti-inflammatory factors through progesterone-induced binding factor (PIBF) (28, 29). Placental progesterone, estradiol, and human chorionic gonadotropin (hCG) facilitate blastocyst implantation and spiral artery formation, which are essential early gestational events to sustain pregnancy. These hormones support implantation by recruiting immune cells to the maternal-fetal interface (30–33). Immune cells subsequently secrete angiogenic factors such as transforming growth factor beta (TGF-β) and vascular endothelial growth factor (VEGF) (31) to promote spiral artery formation (34) and placental vascularization.

The influence that placental steroid hormone production exerts across the maternofetoplacental unit—involving inflammatory responses, oxygen/nutrient exchange, and neuroendocrine functioning—cannot be overstated. This review article will describe the current understanding of the *in utero* steroid-related environment associated with ASD and explore its potential role in ASD pathogenesis. Existing literature that is relevant to the prenatal hormone milieu in ASD largely appears to fall within the following four domains: obstetric conditions, fetal programming, sex differential, and steroid-related biomarkers. The literature on each domain will be summarized.

## Obstetric Conditions

### Hypertensive Disorders

Hypertensive disorders during pregnancy, regardless of subset (i.e., chronic, gestational, *de novo* or superimposed preeclampsia), have been correlated with elevated ASD incidence among offspring (13, 14, 35–37). Placental insufficiency is one of the most concerning complications of hypertension during pregnancy (22, 38, 39). Common indicators or complications of placental insufficiency that overlap with ASD risk factors include small for gestational age/*in utero* growth restriction, prematurity, maternal infection, and maternal metabolic syndrome (16, 38–42). Histologic signs of placental insufficiency, such as trophoblastic inclusions, are also more commonly found in placentas of children who develop ASD (43). Steroid hormone dysregulation, altered immune function, and placental insufficiency are intertwined, as maternal serum inflammatory markers (e.g., atypical cytokine profiles, leukocytosis, and elevated platelet counts) are associated with gestational hypertension (44), while the maternal cardiovascular adaption to pregnancy is influenced by placental estrogen and progesterone production (45).

### Gestational Diabetes/Insulin Resistance

Maternal diabetes, both pre-existing and gestational onset, is also an established ASD risk factor (46, 47). While the exact mechanism underlying the relationship between ASD and maternal diabetes is unknown, maternal diabetes can lead to several obstetric complications affecting the mother (e.g., gestational hypertension, pre-eclampsia) and baby (e.g., high or low birth weight, shoulder dystocia, hypoglycemia, hyperbilirubinemia, hypocalcemia, and respiratory distress) (48, 49). These conditions result from the amplification of the typical prenatal metabolic state characterized by hypercortisolemia (50) and insulin resistance (51, 52). During uncomplicated pregnancies, relative maternal hypercortisolemia and insulin resistance facilitate adequate transfer of nutritional resources from mother to baby. As pregnancy progresses, a developmental switch prompted by rising fetal cortisol synthesis shifts resource allocation from tissue proliferation to maturation (53). In maternal diabetes, excess glucose supply to the fetus causes higher fetal insulin production to maintain glucose homeostasis, stimulating fetal overgrowth and delaying lung maturation (54). Diabetes during pregnancy also leads to higher placental release of pro-inflammatory cytokines (e.g., leptin, tumor necrosis factor-α (TGF-α), and interleukins) (55), with the potential consequence of reducing oxygen diffusion across the placenta by enhancing placental thickening (48).

### Pre-pregnancy Obesity/Gestational Weight Gain

Epidemiologic studies identify increased ASD risk associated with pre-pregnancy obesity (46) and/or gestational weight gain (2, 12, 56). Obesity promotes both inflammation and endocrine dysfunction, perturbing the prenatal environment. As a pro-inflammatory state, obesity contributes to elevated lipids, leptin, and IL-6 during pregnancy (57). The bioavailability and synthesis of estrogens are impacted through the endocrine and metabolic function of adipose tissue (58). Furthermore, studies exploring the *in utero* steroid environment, primarily as it relates to hormone-sensitive cancer risk among offspring, have found elevated maternal serum estrogen, progesterone, and testosterone levels in pregnancies characterized by higher weight gain (58–61). Because the gestational weight gain associated with increased ASD risk (12) is not clinically relevant from an obstetric perspective (i.e., about three pounds), its link with ASD may relate to a shared etiology rather than a cause and effect relationship.

### Maternal Stress

Epidemiologic studies have also found that heightened maternal stress during the 2nd trimester increases fetal vulnerability to adverse outcomes such as shortened gestational age, preterm birth, low birth weight, and small for gestational age (62). ASD has also been associated with intense maternal stress (e.g., life events and hurricanes) during this gestational window (5, 6, 63).

## Fetal Programming

Fetal programming, a concept also referred to as the “developmental origins of health and disease” hypothesis, explains that *in utero* disruption during critical developmental periods can relay health consequences to the fetus that persist throughout adulthood (64–68). Originally bolstered by epidemiological findings describing regional overlap in areas with high infant mortality and coronary heart disease (69), there has been increased recognition that *in utero* phenomena can lead to a wide range of chronic conditions. Supporting evidence includes the well-established relationship between prenatal exposure to maternal metabolic conditions [e.g., hypertension (70), diabetes (71–73), obesity (74–76)] and chronic metabolic disorders that begin in adolescence and adulthood (77, 78). Pre-pregnancy maternal obesity has been linked to poorer metabolic, endocrine, cardiovascular, and neurodevelopmental outcomes in offspring (79). Even after adjusting for pre-pregnancy obesity, maternal metabolic conditions such as gestational diabetes remain associated with increased risk of cardiovascular disease (80), obesity (81), type 2 diabetes (82), and early childhood metabolic syndrome development (83) among offspring.

Fetal programming of adolescent/adult metabolic disorders may occur through epigenetic changes (84). Telomeres are repetitive DNA tracts that protect against excessive chromosomal degradation (85). Shortened telomeres occur in fetuses exposed to gestational diabetes and are associated with higher cardiometabolic disease risk in adults (86, 87). Pre-eclampsia has been shown to induce epigenetic changes in offspring through decreased DNA methylation of *IGF2* (88), a mediator of cell proliferation and apoptosis (89). Aside from its influence on postnatal growth, aberrant *IGF2* expression is linked to subsequent development of hypertension, diabetes, and other metabolic disorders (88).

The proposed basis for fetal programming includes calibration of fetal regulatory systems in response to intrauterine nutrient availability to optimize extra-uterine survival. The subsequent mismatch between pre- and postnatal resources—whether involving excess or scarcity—predisposes offspring to develop one or more metabolic disorders. Fetal programming serves as a potential mechanism through which stress, metabolic disturbances, inflammation, and steroid dysregulation during pregnancy could predispose offspring to ASD. Altered fetal hypothalamic-pituitary-adrenal (HPA) axis development falls within the concept of fetal programming (90–92).

### HPA Axis Functioning in ASD

The HPA axis modulates neural, endocrine, and immune responses to stress to maintain homeostasis (93). While HPA axis plays a critical role in coordinating short-term physiological stress responses (94), HPA axis dysregulation has been implicated in several psychological and physiological disorders (95–100). Some children with ASD demonstrate signs of HPA axis dysregulation (101–106) such as altered circadian rhythms (107) and abnormal cortisol stress responses (105). Collectively, these findings raise the possibility that aberrant HPA axis functioning in some ASD individuals may have originated during fetal life through fetal programming.

### Fetal HPA Axis Maturation

Understanding fetal HPA axis development in the 2nd trimester (i.e., 13th to 26th week gestation) provides a context in which to interpret the association between ASD and steroid hormone levels (whether measured in maternal serum or amniotic fluid) during this gestational window. By the 12th week of gestation, the fetal hypothalamus releases corticotropin-releasing hormone (CRH) to signal the anterior pituitary gland to release adrenocorticotropic hormone (ACTH) (108–110). ACTH stimulates the fetal zone of the adrenal gland to synthesize dehydroepiandrosterone (DHEA) (110) and its conjugated form, dehydroepiandrosterone sulfate (DHEAS) (111). Although the fetal HPA axis is active by 12–18 weeks gestation (112), fetal adrenal gland development lags behind that of the hypothalamus and anterior pituitary. The fetal adrenal gland does not typically develop *de novo* cortisol synthesis capacity—referred to as fetal HPA axis maturation—until around 23–24 weeks gestation (91, 113–115). Because cortisol promotes the physiologic shift from somatic growth to organ maturation [e.g., lungs (116), gut (117), liver (116, 118)], fetal HPA axis maturation is essential for extra-uterine survival (119). Under duress, fetal HPA axis maturation can occur as early as 20 weeks gestation (113). This may be induced by increased placenta estradiol production (120).

### The Placenta's Role in Fetal HPA Axis Maturation

**Figure 1:**
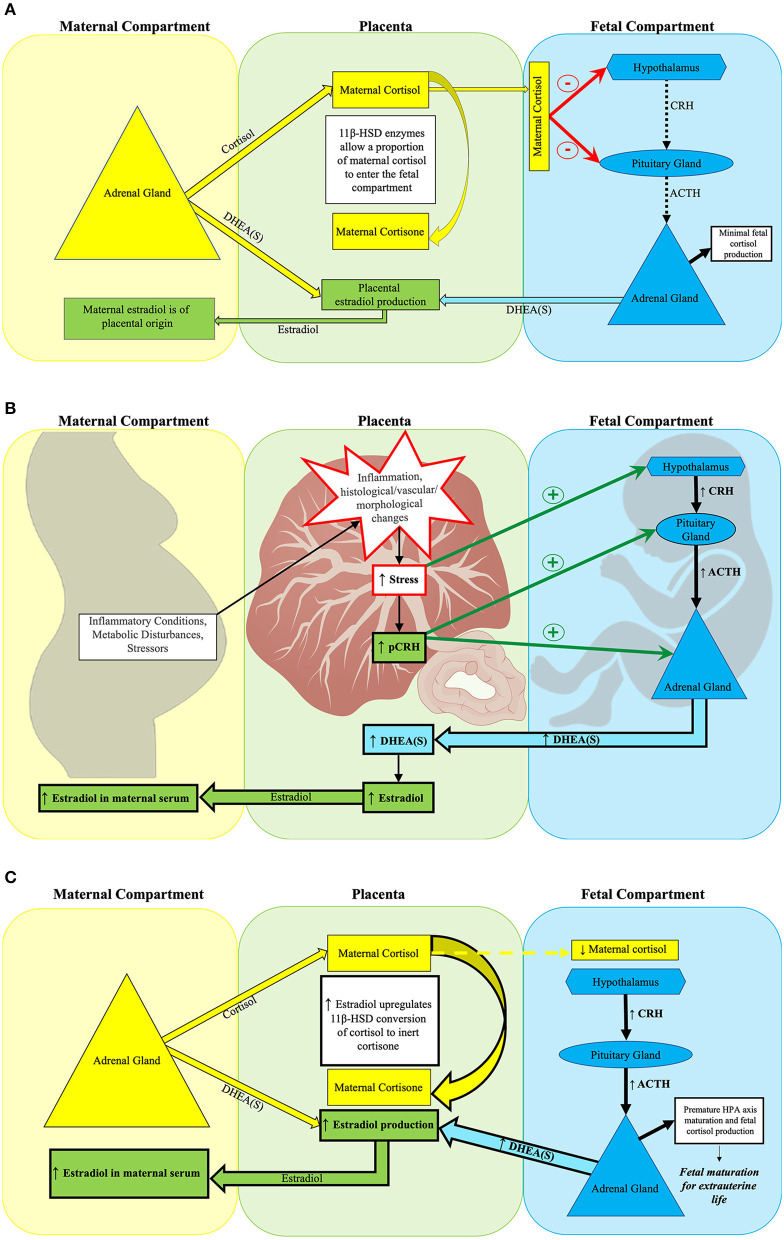
Placental estradiol and the fetal HPA axis at mid-gestation. **(A)** Normal fetal HPA axis functioning in the setting of typical placental estradiol activity at mid-gestation. **(A)** Depicts normal suppression of fetal HPA axis activity during mid-gestation by maternal cortisol. The placental glucocorticoid barrier contains 11β-HSD enzymes, which control fetal exposure to maternal cortisol (110) by converting most of the maternal cortisol entering the placenta to its inert form, cortisone. While fetal cortisol levels are 5–10 times lower than maternal cortisol levels (127), the maternal cortisol that enters the fetal compartment suppresses fetal HPA axis activity through negative feedback on the fetal hypothalamus and pituitary gland (112). Typically, the fetal adrenal gland has not yet developed *de novo* cortisol synthesis capacity at this point in gestation (124). Rather, the fetal adrenal gland primarily produces DHEA(S) when stimulated by ACTH or pCRH. Fetal DHEA(S), along with maternal DHEA(S), subsequently serves as the substrate for placental estradiol production (115, 128, 129). The placenta shunts over 90% of estradiol produced into the maternal circulation, thus maternal serum estradiol levels by mid-gestation reflect placental estradiol production (130). **(B)** Obstetrical adversity increases placental estradiol production at mid-gestation. Maternal adversity [e.g., stressors (131), inflammation (132–134), and metabolic disorders (135–138)] can increase placental estradiol production by stimulating pCRH release through disrupting placental structure and function. Subsequently, fetal stress increases, which activates the fetal HPA axis. Both pCRH and fetal HPA axis activation increase fetal adrenal DHEA(S) synthesis, leading to higher placental estradiol production. **(C)** Early fetal HPA axis maturation precipitated by excess placental estradiol activity at mid-gestation. Estradiol acts on 11β-HSD placental glucocorticoid barrier enzymes to increase conversion of maternal cortisol to inert cortisone, thereby reducing the amount of maternal cortisol entering the fetal compartment (139). Less maternal cortisol in the fetal compartment reduces its negative feedback on the fetal hypothalamus and pituitary gland. The relative absence of negative feedback precipitates fetal HPA axis maturation, which is characterized by the onset of *de novo* cortisol synthesis capacity by the fetal adrenal gland (125). Higher placental estradiol production leads to elevated maternal serum estradiol concentrations.

Placental estradiol synthesis promotes fetal HPA axis maturation through multiple mechanisms. Estradiol influences cortisol transfer from the mother to fetus to establish a maternal-fetal cortisol gradient through its actions on placental 11β-hydroxysteroid dehydrogenase (11β-HSD) activity (121). 11β-HSD converts active maternal cortisol into inert cortisone. This gradient serves to protect the fetus from maternal cortisol overexposure (122, 123). Maternal cortisol in the fetal compartment exerts negative feedback on the fetal hypothalamus and anterior pituitary gland. As pregnancy progresses, 11β-HSD activity increases, less maternal cortisol reaches the fetal compartment, and negative feedback to the fetal HPA axis is reduced (115, 122, 124–126). The subsequent increase in fetal HPA axis activation coincides with the emergence of the fetal adrenal's *de novo* cortisol synthesis capacity (125) leading to fetal HPA axis maturation (see Figure 1). The placenta synthesizes estradiol from its precursor DHEA(S) which is derived from both fetal and maternal adrenal glands (124, 140). Excess fetal DHEA(S) supply can drive increased placental estradiol synthesis (110, 124, 141, 142). Because the placenta shunts over 90% of the estradiol it produces into the maternal circulation, maternal serum estradiol levels reflect placental estradiol synthesis (130).

The placenta also influences fetal HPA axis maturation through its production of CRH (pCRH). pCRH stimulates fetal adrenal DHEA(S) production by (1) signaling fetal pituitary ACTH release (143), (2) heightening fetal adrenal gland sensitivity to ACTH (144), and (3) stimulating DHEA(S) producing fetal adrenal cells directly (145, 146). By increasing fetal DHEA(S) synthesis, pCRH facilitates increased placental estradiol production. As estradiol acts on placental barrier enzymes that modulate fetal cortisol exposure, pCRH can indirectly facilitate precocious HPA axis maturation by decreasing negative feedback from maternal cortisol. pCRH also directly promotes adrenal *de novo* cortisol synthesis (147, 148). Following its maturation, activation of the fetal HPA axis signals adrenal production of both DHEA(S) and *de novo* cortisol.

### Inflammation and Hypoxia Stimulate Early Fetal HPA Axis Maturation

Excess inflammation triggered by infection, tissue injury, autoimmunity, or disruption in immunogenic tolerance to the fetus (149) can provoke early fetal HPA axis maturation through increased fetal HPA axis activity (115). Likewise, hypoxia can also stimulate fetal HPA axis maturation through a similar mechanism (150). Considering estradiol's role in strengthening the maternal-fetal cortisol gradient, estradiol serves as a feasible intermediary through which inflammation and hypoxia contribute to precocious fetal HPA axis maturation.

Furthermore, pCRH synthesis is upregulated in response to signifiers of an adverse intrauterine environment, such as elevated cortisol levels, pro-inflammatory cytokines, catecholamines, and decreased uterine blood flow (115, 151, 152). As pCRH promotes ACTH release from the fetal pituitary gland (143) and expression of cortisol synthesizing enzymes (147), elevated pCRH from *in utero* stress can serve as an additional mechanism through which the fetal HPA axis matures precociously (153).

### Sex Differential in ASD

One of the strongest risk factors for developing ASD is male sex. ASD prevalence in males is 3 to 4 fold higher than in females (154–156). In the absence of intellectual disability, this ratio increases further to 7:1 (157). Baron-Cohen et al. (158) has proposed the “extreme male brain” theory to explain the significant sex differential in ASD incidence. This theory suggests that excess prenatal androgen exposure contributes to the development of autistic traits in offspring by amplifying traits thought to be more typical of males, such as systemization, while diminishing traits commonly associated the femininity, such as empathy (158). Sex steroid hormones have long been thought to exert profound effects on the sexual dimorphism of the brain (159, 160); however, the exact mechanism has yet to be fully understood.

The “aromatization” hypothesis proposes that the roles of estradiol and testosterone in fetal neurodevelopment are intertwined (161), as masculinization of the rodent fetal brain is dependent upon the conversion of testosterone to estradiol by the enzyme aromatase (162, 163). In primates, reliance on this mechanism to explain masculinization of the human fetal brain remains unproven. Unlike primates, alpha-fetoprotein in rodents has a high binding affinity to circulating estradiol thereby sequestering estradiol and preventing it from entering and masculinizing the fetal rodent brain (164). Therefore, only the sex steroids produced by the fetal rodent influence brain masculinization. The assumption that estradiol's masculinizing role in the rodent fetal brain could be extrapolated to the human fetal brain is significantly flawed because it fails to account for the substantial difference between these mammals in regards to *in utero* estradiol sequestration by alpha-fetoprotein.

Zhao et al. (165) describes a multiple threshold liability model to explain the sex difference in ASD risk. This model theorizes that females require a higher genetic mutation load relative to their male counterparts to develop ASD thus contributing to ASD's male predominance. Werling and Geschwind (157) speculate that these mutations may interact with androgen-related mechanisms in families with affected females, as prior studies have found an association with ASD and genes that modulate sex-steroid function (166–168). As steroid hormone receptors exert epigenetic effects through DNA methylation and histone acetylation (169–172), it is possible that epigenetic modifications in response to *in utero* sex-steroid hormone exposure affect sex-specific neurodevelopmental processes.

### Sex-Specific Fetal and Placental Responses to Adversity

**Figure 2:**
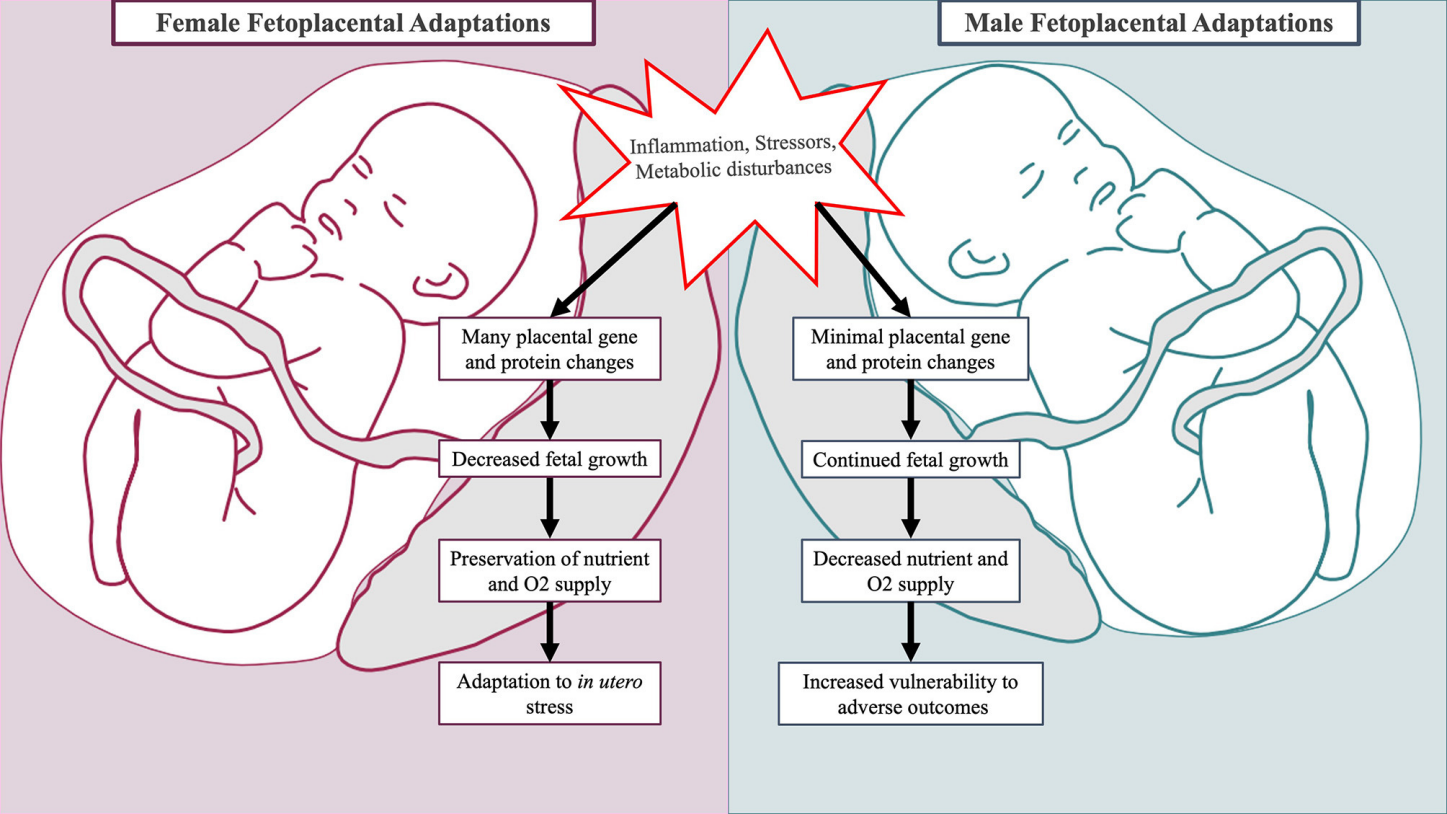
Sex-specific fetal and placental adaptations to maternal adversity. Placentas of male and female fetuses respond differently to mild forms of maternal adversity. In the placenta of female fetuses, multiple changes in glucocorticoid barrier enzyme activity, gene expression, and protein synthesis occur leading to decreased growth (176, 177). This is advantageous as it preserves fetal oxygen and nutrient delivery. In the placenta of male fetuses, minimal changes in gene and protein expression occur, and the male fetus continues to grow incurring increased vulnerability to adverse outcomes (176).

Fetal growth, development, and HPA axis programming in the setting of obstetric adversity differ by fetal sex (173–175). These sex-specific responses serve as additional mechanisms in which to consider male ASD predominance (see Figure 2). As the placenta is derived from extra-embryonic tissues, the placenta has the same sex as the fetus (178). Evidence suggests that the placentas of male and female fetuses differ in response to adverse prenatal environments through modulation of steroid pathways, placental genes, and protein synthesis (176). Placental growth and structure differ by sex, with male placentas being smaller in size but more efficient at nutrient and oxygen delivery (179, 180). Fetal growth depends upon the limited capacity of the maternal-placental interface to deliver oxygen and nutrients. Thus, greater placental efficiency among males precipitates faster somatic growth while increasing vulnerability to *in utero* perturbations (179, 181). This may have deleterious neurodevelopmental consequences, as fetal brain development relies on the availability of oxygen and nutrients such as fatty acids, glucose, and amino acids (131, 182, 183). In contrast, female placentas may have superior ability to buffer and adapt to suboptimal prenatal conditions (180).

Newborns differ by sex in regards to birth weight, morbidity, and mortality. This is attributable at least in part to sex-specific adaptions that regulate the balance between fetal growth and extra-uterine survival (184, 185). In the setting of mild physical adversity (i.e., chronic maternal asthma), changes in placental 11β-HSD glucocorticoid barrier enzyme activity, gene expression, and protein synthesis occur and coincide with reduced female fetal growth (176, 177). This prepares the female fetus for future adverse events through preservation of oxygen and nutrient delivery. In pregnancies complicated by mild pre-eclampsia, male and female fetuses differ in growth progression mediated by differential fetal microvascular (186) and placental inflammatory cytokine responses (187). In the setting of maternal hyperglycemia, males demonstrate greater hyperglycemic growth stimulation (188) and higher incidences of respiratory distress (175). The sex hormones estradiol and testosterone stimulate opposing processes on fetal lung development: estradiol facilitates surfactant production while testosterone promotes lung tissue proliferation at the expense of lung maturation (189). Collectively, sex-specific responses to obstetrical adversity leave males less physiologically prepared for survival, particularly if birth were to occur prematurely (176).

A growing body of evidence demonstrates a link between placental pathology and ASD. Anderson et al. (43) found increased trophoblastic inclusions in the placentas of fetuses who later developed ASD. Trophoblastic inclusions, a histological finding that results from atypical growth and folding of the placenta (190), are more common in genetically atypical gestations (191–194). Furthermore, studies of high ASD risk cohorts (defined by having at least one older sibling with ASD) also found increased placental trophoblastic inclusions (195) in addition to altered placental morphology (196) and placental chorionic surface vascular networks (197). The specific morphological changes found, such as increased thickness and roundness, may reflect decreased ability to adapt to variations in the prenatal environment (196). Notably, variations within the placental chorionic surface vascular networks may be the result of atypical vasculogenesis and angiogenesis (197). Several conditions associated with ASD, such as pre-eclampsia (198), intrauterine growth restriction (41), and pre-term birth (16) are also attributed to placental vascular abnormalities (199–201). In ASD individuals, Straughen et al. demonstrated an association between placental inflammation, maternal vascular malperfusion, and ASD (9) that occurred more prominently among males.

Sex-specific placental signaling and epigenetic phenomena in fetal adaption to nutrient availability and maternal stress are exemplified by the X-linked gene O-linked N-acetylglucosamine transferase (OGT) (131, 202). OGT induces glucose-sensitive epigenetic changes that influence immune responses, steroidogenic activity, and fetal development. Because placental X inactivation spares OGT (20) such that the female placenta has two active gene copies while the male placenta has only one, OGT expression and interaction with steroid receptors demonstrate sex-specificity (203, 204). This provides an additional potential mechanism through which maternal metabolic conditions (i.e., hyperglycemia, insulin insensitivity) could influence the sex-differential observed in ASD (20).

## Steroid-Related Biomarkers in ASD

*In utero* steroidogenesis occurs through the well-coordinated interactions among the mother, placenta, and fetus (21, 22). The placenta is the main orchestrator of adaptions by the maternofetoplacental unit of steroidogenic activity in response to *in utero* and external environmental cues (205). As the transcriptome of the placenta changes throughout pregnancy (206), expression of placental genes involved in steroid hormone regulation consequentially shift (205). This section is a review of steroid-related biomarkers that have been associated with ASD accompanied by a brief summary of their potential connection to ASD.

### Androgens

Multiple studies have demonstrated associations between ASD/ASD traits and amniotic fluid androgen levels (i.e., androstenedione and testosterone) (207–212) supporting the “extreme male brain” theory for ASD's etiology proposed by Baron-Cohen et al. (158). Amniocentesis is typically performed between the 15th and 20th week of pregnancy, overlapping the critical gestational window when the male fetus produces peak amount of androgens to promote genital differentiation (213). This testosterone surge occurs between 11 and 17 weeks gestation (214).

Elevated androgen levels (androstenedione and testosterone) in amniotic fluid collected from pregnancies with male offspring correlated with later ASD diagnoses (212). Decreased eye contact (207), poorer quality of social relationships (211), increased restricted interests (211), reduced empathy (210), and higher autism trait scores (208, 209) have also been associated with elevated amniotic fluid androgen levels. Researchers conducting these amniotic fluid studies applied ASD trait severity measures that they developed, including the Quantitative Checklist for Autism in Toddlers (Q-CHAT) (215), the Childhood Autism Spectrum Test (CAST) (216), and the Child Autism Spectrum Quotient (AQ-Child) (217). In contrast, Kung et al. (218) found no relationship between autistic traits and amniotic testosterone levels in pregnancies resulting in typically developing children using the CAST. Elevated amniotic androgen levels associated with ASD found by Baron-Cohen et al. (212) also did not appear to persist in a re-analysis published in Baron-Cohen et al. (219).

Maternal serum has also been analyzed to investigate the relationship between prenatal androgen levels and the emergence of ASD (19). Maternal serum drawn during early 2nd trimester did not identify significant associations between maternal androgen levels and ASD among offspring (19). Although gestational timing for the maternal serum and amniotic fluid collections overlap, differences in study findings between Baron-Cohen et al. (212) and Bilder et al. (19) may result in part from differences in study purposes and designs. For the maternal serum study, investigators were particularly interested in measuring steroid-related biomarkers as potential intermediaries between ASD outcome and the effects of obstetrical conditions associated with steroid dysregulation (i.e., hypertension and diabetes) which they referred to as “prenatal metabolic syndrome” (PNMS). As such, both the ASD case and non-ASD control groups were enriched (by 50%) for the presence of PNMS exposure. Extrinsic testosterone exposure to the fetus is measurable in maternal serum, though fetal testosterone production cannot be measured in maternal serum because androgen movement across the placenta is generally considered unidirectional from mother to fetus (220, 221). Testosterone levels in amniotic fluid, however, reflect both fetal testosterone exposure and production as the amniotic fluid collected is composed primarily of fetal urine.

In Park et al. (222), no association was found between androgen levels (testosterone, androstenedione, and DHEA) in umbilical cord blood and autistic traits at 12 and 36 months of age among the Early Autism Risk Longitudinal Investigation (EARLI) cohort using the Autism Observation Scale for Infants (AOSI) and Social Responsiveness Scale (SRS), respectively. The EARLI cohort were younger siblings of children with ASD. Several other studies have also failed to demonstrate an overall relationship between umbilical cord testosterone levels and ASD traits (223–225). Unlike the amniotic fluid and maternal serum studies described above, umbilical cord blood samples represent prenatal testosterone exposure at the time of parturition, far after the fetal testosterone surge occurs (214). Upon subdividing the sibling cohort into multiple groups based on proband and participant sex, umbilical cord blood testosterone levels correlated with ASD traits among siblings of female probands. The authors attributed their findings to the multiple threshold liability model (157, 165) suggesting a higher genetic load required for females to develop ASD.

Meconium androgen levels were also measured in the EARLI cohort (226). Findings demonstrated a positive correlation with some androgen levels (i.e., unconjugated testosterone and total DHEA) and SRS scores overall. Following stratification of the cohort by participant and proband sex, various associations were found between androgen levels and ASD traits at 12 and 36 months of age. Meconium begins accumulating in the 13th week of gestation, with the highest volume produced between 28 and 34 weeks gestation (227). As meconium represents cumulative exposures throughout gestation (228), it is difficult to link meconium steroid concentrations to a specific gestational time period of exposure.

Fetal testosterone in males is predominantly produced by the Leydig cells of the testes beginning around 8 weeks gestation (229). Placental human chorionic gonadotropin (hCG), in tandem with luteinizing hormone (LH) produced in the fetal pituitary gland, stimulates testosterone synthesis by the testes during early gestation (214, 230–233). Interestingly, LH and hCG share a common receptor transcribed from a single gene (234). Placental hCG production rapidly increases following implantation until it reaches its apex at the end of the first trimester (235). Following this, hCG levels progressively reach a nadir by 18–20 weeks gestation (236–238). As hCG levels decline, LH levels rise. From 8 to 24 weeks of gestation, the testosterone level in males substantially exceeds that of females (239), which is believed to impart neurodevelopmental effects resulting in sex-specific behavioral differences (240). However, the timing and mechanism in which this occurs in the human fetal brain has not been definitively determined. While the fetal adrenal gland can also synthesize testosterone, the amount is negligible (241, 242) and its contribution to amniotic fluid androgen levels is not clearly established.

### hCG

Aside from stimulating testosterone synthesis, hCG serves a variety of functions, including pregnancy maintenance through stimulation of the corpus luteum to secrete progesterone (129, 243). Additionally, hCG has been associated with angiogenesis, mediation of immune tolerance, umbilical cord development, myometrial contraction suppression, and fetal organ growth and differentiation (244–246). To our knowledge, only one study has directly examined 2nd trimester maternal serum hCG levels in relation to ASD risk. Windham et al. (247) found a U-shaped relationship (i.e., both higher and lower levels) between hCG levels associated with increased ASD risk among offspring, particularly in males.

Elevated hCG levels during the 2nd trimester have also been linked to complications such as preeclampsia (248–250), intrauterine growth restriction (IUGR) (248), pre-term delivery (248, 250, 251), low birth weight (250), and fetal death (250, 251). As the placenta secretes hCG in response to stress hormones (252), an adverse prenatal environment may contribute to steroid dysregulation in ASD through elevated hCG exerting its influence on testosterone synthesis in male fetuses. This proposed mechanism of aberrations within the fetal steroid hormonal milieu aligns with findings of elevated 2nd trimester amniotic fluid testosterone levels associated with ASD described above. Interestingly, hCG can also stimulate fetal adrenal DHEA(S) secretion during the 2nd trimester (253). As DHEA(S) is a substrate for placental estradiol synthesis, abnormalities in 2nd trimester estradiol and testosterone linked to ASD development may share a common genesis involving the placental response to adversity.

### Estrogens and Progesterone

While prior investigations have focused on androgens, recent research studies have explored the potential role that estrogen and progesterone may play in the development of ASD. Bilder et al. (19) found significantly higher estradiol levels in early 2nd trimester maternal serum associated with ASD among offspring. As described above, both ASD and comparison cohorts were enriched for PNMS exposure. Along with higher estradiol levels, lower SHBG levels were also identified, suggesting the potentiation of estradiol activity in pregnancies associated with ASD as SHBG binds biologically active estrogens rendering them inert (254). Estradiol, rather than estriol, was selected as the estrogen of interest for this study because maternal estradiol levels represent placental estrogen activity more accurately through estradiol's substantially higher potency (255), longer estrogen receptor binding duration (256), and higher maternal serum concentrations (257, 258). Because maternal serum estradiol levels result from, and reflect, placental estradiol production by this gestation window (130), Bilder et al. (19) interpreted these results as indicating increased ASD risk associated with elevated placental estradiol production and activity. Higher placental estradiol production could result from greater fetal steroidogenic activity, and increased placental estradiol activity could facilitate premature fetal HPA axis maturation in the early 2nd trimester. Bilder et al. (19) did not find a significant association between serum progesterone concentrations and ASD risk.

Baron-Cohen et al. (219) also identified higher estrogen levels (i.e., estradiol, estriol, and estrone), along with increased progesterone levels, in 2nd trimester amniotic fluid of male offspring with ASD relative to controls. Notably, elevated estradiol was the most significant predictor of subsequent ASD development. Baron-Cohen et al. (219) attributes the link between higher amniotic estrogens, progesterone, and ASD among offspring to increased fetal steroidogenic activity in ASD's etiologic pathway.

Elevated ASD risk associated with higher prenatal estrogen levels in maternal serum (19) and amniotic fluid (219) contrast with prior study results from Windham et al. (247), which demonstrated lower unconjugated estriol levels in 2nd trimester maternal serum. Estriol is a weak estrogen produced exclusively during pregnancy and requires fetal liver enzyme activity in its synthesis pathway (257, 258). Estriol levels in Windham et al. (247) were initially measured as a component of a prenatal integrated screening test for aneuploidy and neural tube defects. Low maternal serum estriol levels have been used for decades as a clinical indicator for obstetric complications (259), such as placental insufficiency (260), fetal growth restriction (261), pre-eclampsia (262), preterm birth (247), low birth weight (263), and pregnancy loss (260). Windham et al. (247) attributes study findings to the involvement of hormones in the development of ASD through their influence on fetal development and signaling activity in the CNS and immune system.

### Cortisol

Baron-Cohen et al. (212) identified elevated cortisol levels in the amniotic fluid collected during the pregnancies of male offspring who developed ASD, though this finding was not replicated in Baron-Cohen et al. (219). However, Baron-Cohen and colleagues (212, 219) conclude that the findings from both studies support the presence of increase fetal steroidogenic activity in ASD. The significant diurnal variation in serum cortisol concentrations preempts its use as a biomarker in banked maternal serum samples from most large obstetric studies because standardization of serum collection times for these studies is typically not implemented.

### Sex Hormone-Binding Globulin (SHBG)

Bilder et al. (19) found an inverse relationship between maternal serum SHBG levels and risk for diabetes/hypertension exposure and ASD. The highest SHBG levels were measured for the cohort with neither PNMS exposure nor ASD. Conversely, the lowest SHBG levels occurred in the cohort with both PNMS exposure and ASD. In addition to binding estrogens and testosterone, SHBG also serves as a serum biomarker for insulin sensitivity that supersedes its relationship with sex hormones regardless of sex, age, and pregnancy status (264). Even among pregnancies without clinical PNMS manifestations, SHBG levels were lower in the ASD cohort compared to either non-ASD cohorts, suggesting the presence of exposure to a subclinical metabolic condition during gestation in these offspring who developed ASD. Unlike most serum biomarkers for insulin sensitivity, SHBG measures require no specific collection time (e.g., time of day or proximity to caloric consumption) to ensure meaningful results interpretation. SHBG has been studied as a predictive 1st and early 2nd trimester biomarker for the emergence of gestational diabetes (265–267). Thus, SHBG is a useful biomarker for investigating maternal insulin sensitivity as a prenatal risk factor within maternal serum samples banked for non-specific purposes.

### “Estimated Fetal DHEA”

As a *post hoc* analysis, Bilder et al. (19) calculated a value from maternal serum measurements of estradiol, DHEA, and DHEAS to estimate the relative contribution by the fetal adrenal gland to the DHEA substrate supply for placental estradiol production (EF-DHEA). The area under the curve for EF-DHEA used to predict ASD among offspring exceeded those of its measured components. This was interpreted to indicate that the relationship between elevated maternal serum estradiol and increased ASD risk may in part be driven by excess fetal adrenal activity in early 2nd trimester. The EF-DHEA calculation was created specifically for this study and has not been validated in animal models nor have these findings been replicated. However, EF-DHEA findings are consistent with the amniotic fluid results, particularly elevated cortisol, from Baron-Cohen et al. (212) demonstrating an association between ASD among offspring and increased fetal steroidogenic activity during this gestational window.

### Synthesis of Pertinent Biomarker Findings

**Figure 3:**
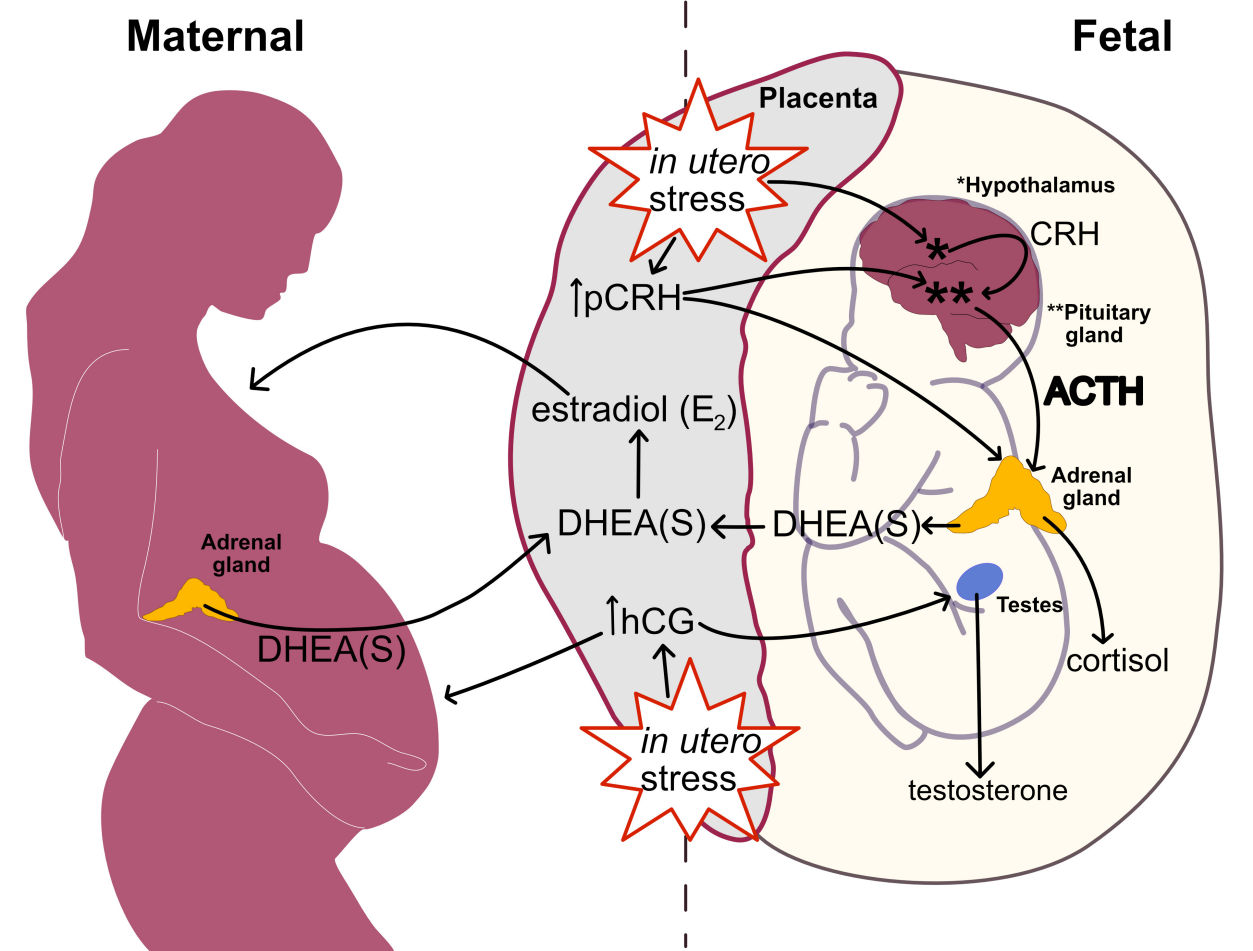
Linking *in utero* stress to increased fetal steroidogenic activity and ASD biomarkers at mid-gestation.

Figure 3 links heightened *in utero* stress from inflammation, stressors, and metabolic disturbances to perturbation within the prenatal hormone milieu. Through pCRH stimulation, the placenta upregulates fetal HPA axis activity in response to *in utero* stress. Subsequently, the fetal adrenal glands increase DHEA(S) synthesis leading to elevated placental estradiol production. Higher placental estradiol and pCRH production promotes HPA axis maturation denoted by fetal adrenal *de novo* cortisol synthesis. In response to *in utero* stress, the placental also increases hCG production which stimulates fetal gonadal testosterone synthesis.

## Discussion

This review provides an up-to-date synopsis of the current evidence and supported theories regarding the role that the *in utero* steroid environment may play in ASD pathophysiology. Considering placental steroid hormone biosynthesis, metabolism, and transport (205), prenatal steroid dysregulation may be attributed to disruptions in vital components of placental structure and function in response to an adverse maternofetoplacental environment. In particular, stress, inflammation, and metabolic abnormalities can contribute to morphological and functional placental changes that affect nutrient and oxygen exchange, the protective transplacental barrier, and hormone synthesis (268, 269).

Obstetric conditions associated with ASD that may contribute to an adverse prenatal environment include hypertensive disorders of pregnancy (i.e., chronic, gestational, *de novo* or superimposed preeclampsia) (13, 14, 35–37), maternal immune dysfunction (5, 7–9), pre-existing/gestational diabetes (3, 10), pre-pregnancy obesity (11), gestational weight gain (12), and PCOS (4, 15). Notably, common indicators or complications of placental insufficiency also overlap with ASD risk factors, including small for gestational age/*in utero* growth restriction, prematurity, maternal infection, and maternal metabolic syndrome (16, 38–42). As the placenta is critical for fetal growth and development, adverse prenatal environments that impact the placenta may increase fetal stress and influence fetal programming, thereby contributing to ASD etiology.

The placenta shares the sex of the fetus as it is derived from extra-embryonic tissues (178). Numerous studies have documented that placentas of male and female fetuses differ in response to adverse prenatal environments (270–273). Specifically, placental signaling and gene expression influence sex-specific differences in fetal adaption to the *in utero* environment related to nutrient availability, maternal stress, and immune response (131, 202). In the setting of mild adversity, ongoing somatic growth of male fetuses confers increased vulnerability to subsequent adverse events (176, 177) that can influence neurodevelopment. Additionally, evidence of placental pathology appears more prominent from pregnancies of male offspring with ASD compared to those of their female counterparts. Further investigation of placental pathology in ASD is needed, particularly as it relates to fetal/placental sex differences.

Second trimester serves as a critical time for fetal steroidogenic activity in regards to fetal HPA axis maturation. Multiple ASD epidemiologic studies highlight the 2nd trimester as the period of greatest fetal vulnerability to stressful maternal life events (5, 6, 63). Bilder et al. (19) and Baron-Cohen et al. (212, 219) interpret the link between higher estrogen levels and ASD risk as an indicator of elevated fetal steroidogenic activity in the 2nd trimester of offspring who develop ASD. While Baron-Cohen et al. (212, 219) conceptualize fetal steroidogenic activity more broadly, particularly in regards to fetal androgen production, Bilder et al. (19) focuses on excess fetal adrenal activity and premature fetal HPA axis maturation. By strengthening the maternal-fetal cortisol gradient, placental estradiol reduces the amount of maternal cortisol in the fetal compartment (139) thereby easing maternal cortisol suppression of the fetal HPA axis and promoting its maturation. Fetal steroidogenic activity is enhanced by CRH released by the fetal hypothalamus and placenta (124, 145, 274–276). CRH production is stimulated by stress, inflammation, and hypoxia providing a mechanism through which obstetrical adversity could contribute to increased fetal steroidogenic activity and early HPA axis maturation (115, 150, 277).

The relationship between higher 2nd trimester estrogen levels and increased ASD risk is intriguing as estrogen is widely seen as an indicator of maternal and fetal health. Estradiol acts to preserve fetal viability during *in utero* stress through suppression of inflammation (278, 279) and uterine artery dilation that increases oxygen and nutrient supply to the fetus (280, 281). Estradiol also mediates several critical factors in neurodevelopment providing an overall neuroprotective benefit (162). From the perspective of fetal viability, excess estradiol in the early 2nd trimester can stimulate precocious fetal HPA axis maturation in preparation for extrauterine life. As described above, low—rather than high—maternal serum estrogen levels predict several conditions related to maternal and fetal adversity. Therefore, it is possible that elevated estradiol in early 2nd trimester maternal serum and amniotic fluid may be the result of a compensatory rather than primarily pathologic mechanism. However, it must be noted that the historical obstetric practice of using a synthetic estrogen to foster healthy pregnancies was quite misguided. The use of the synthetic estrogen diethylstilbestrol (DES) in pregnant women during the mid-1900's was halted when exposed adolescent and young adult female offspring were found to develop a rare type of cancer, vaginal adenocarcinoma (282). Subsequent studies have found transgenerational adverse effects of DES exposure, as reproductive tract and immunologic abnormalities have occurred in exposed male and female offspring (283). Grandchildren of exposed women were also found to have a higher incidence of ADHD (284). To our knowledge, there have been no studies published that report on an epidemiologic investigation of ASD risk associated with DES exposure. Although DES exposure differs in many respects from increased placental estrogen synthesis, such a study could improve understanding of estrogen's potential role in ASD's etiology.

An early theory connecting the prenatal steroid environment with ASD conceptualizes ASD as an extreme manifestation of male cognitive traits and implicates excess *in utero* androgen exposure in ASD's etiology (158). Although elevated amniotic testosterone levels were associated with the presence of ASD traits in most (207–211), but not all studies (218), results regarding ASD diagnosis and testosterone concentrations have been more variable (212, 219). Two amniotic fluid studies and a maternal serum study found no relationship between testosterone and ASD (19, 218, 219). Results from umbilical cord blood and meconium analyses were mixed (222, 226). Disparate findings on androgen biomarkers may be attributable to differences in sample type, gestational timing, ASD outcome measures, and cohort characteristics. Additional investigations are needed which implement multiple sampling strategies and gestational time points in a large obstetrical cohort to clarify the relationship between androgen levels throughout pregnancy and ASD risk.

Overall, prior research findings have established that an altered prenatal environment is a component of ASD pathogenesis. However, the exact process detailing the interaction between diverse prenatal factors and increased ASD risk remains unclear. While several studies link various aspects of perturbations *in utero* to ASD development, to our knowledge, there has been a lack of a unifying paradigm. Based on our current understanding, we propose that disruption within the maternofetoplacental unit initiates a causal sequence resulting in placental changes, thereby influencing fetal programming, steroid hormone modulation, and HPA axis development in ASD.

Factors that contribute to disruption within the maternofetoplacental unit include metabolic disturbances, inflammatory conditions, and stressors. These broad categories are manifested by several obstetric conditions that are associated with ASD development. The placenta reacts to changes *in utero* through adaptations that differ based on sex, which are disadvantageous to male fetuses. Fetal programming is affected, which may have neurodevelopmental implications. Considering the placenta's endocrine functions, steroid hormone modulation is also impacted which could alter HPA axis development and functioning. This may be relevant to HPA axis dysregulation found in some individuals with ASD.

Current literature findings support multidisciplinary research efforts to investigate the manner in which the *in utero* steroid milieu and fetal steroidogenic activity interact with genetic/epigenetic, environmental, inflammatory, and obstetrical ASD risk factors. Studies examining these interaction effects on fetal neuroendocrine development may identify feasible intervention targets that could reduce risk for neonatal morbidity/mortality and childhood neurodevelopmental disabilities. In particular, elucidating the link between the *in utero* steroid environment and immune response may provide an opportunity to optimize both immediate outcomes and long-term neurodevelopmental functioning.

## Author Contributions

WW conceptualized and designed all components of the paper, drafted the initial manuscript, reviewed, and revised the manuscript. SD conceptualized the obstetrical components of the paper, reviewed, and revised the manuscript. DB supported the conceptualization of all components of the paper, reviewed and revised the manuscript. All authors approve the final manuscript as submitted and agree to be accountable for all aspects of the work.

## Conflict of Interest

DB was a consultant for BioMarin Pharmaceutical and Encoded Therapeutics and serves as a consultant and scientific advisory board member for Taysha GTx. The content of this manuscript does not overlap with her work for these companies. The remaining authors declare that the research was conducted in the absence of any commercial or financial relationships that could be construed as a potential conflict of interest.
